# Seeking the first phylogenetic method–Edvard A. Vainio (1853–1929) and his troubled endeavour towards a natural lichen classification in the late nineteenth century Finland

**DOI:** 10.1007/s40656-024-00635-5

**Published:** 2024-11-06

**Authors:** Samuli Lehtonen

**Affiliations:** Biodiversity Unit, University of Turku, 20014 Turku, Finland

**Keywords:** Carl Wilhelm von Nägeli, Ernst Haeckel, Monophyly, Phylogenetic method, Theory of evolution, Lichenology

## Abstract

Edvard August Vainio was a world-renowned Finnish lichenologist. In Finland, however, he was a controversial person due to his strong pro-Finnish political views. Equally disputed was his opinion that systematics should be based on evolutionary theory and phylogenetic thinking. Vainio was familiar with the ideas of the early German phylogeneticists—especially those of Carl Wilhelm von Nägeli – and, applying them, aimed to create an exact method for building a natural classification of lichens already at the end of the nineteenth century. In this respect, Vainio was a true pioneer, as no actual phylogenetic method had yet been developed. In the general spirit of the time, Vainio focused on finding the ancestors of species and other taxa by comparing primitive and derived features of homologous characters. However, Vainio already understood the concept of sister groups in 1880, the identification of which is the basis of all modern phylogenetic research. Nevertheless, the distinctive method developed by Vainio was not so much focused on the construction of a phylogenetic tree, but on revealing the origin of species through the differentiation and fixation of their polymorphic variation. Indeed, Vainio’s species concept is surprisingly similar to the phylogenetic species concepts presented a hundred years later. Although in many ways progressive, Vainio’s views did not influence the development of phylogenetics more widely, but his discussions are nevertheless a valuable source to understanding the early development of phylogenetic theory.

## Introduction

**Fig. 1:**
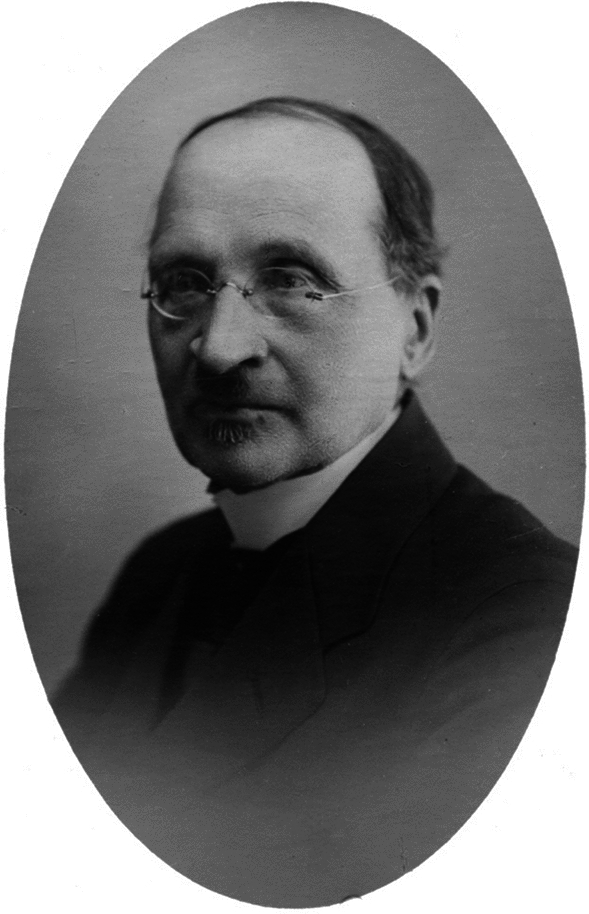
Finnish lichenologist and pre-Hennigian phylogeneticist, Edvard August Vainio (1853–1929)

Edvard August Vainio (1853–1929, until 1876 Lang, 1876–1919 Wainio^1^) was internationally highly esteemed Finnish lichenologist who introduced phylogenetic thinking into Finland in 1880 (Fig. 1). But in Finland, under Russian rule at that time, Vainio’s radical pro-Finnish political views caused him serious problems in the entirely Swedish-speaking academic circles of Finland (Vitikainen, 1998). The language schism was not alleviated by his equally extreme opinion that systematics should be based on evolutionary thinking and phylogenetic reasoning – an opinion which resulted in a total breakup between him and William Nylander (1822–1899), a world famous Finnish lichenologist and Vainio’s early mentor (Vitikainen, 1998).

Before entering into lichen systematics Vainio had completed his licenciate of philosophy degree by defending a thesis on plant geography in 1878 (Linkola, 1934). This thesis (Wainio, 1878a) was the first biological dissertation ever published in Finnish, demonstrating Vainio’s continuous attempts to promote Finnish language in the academia. At the same time, the thesis was a significant contribution towards the Finnish forest and mire site-type classification systems, even though Vainio’s role in the early development of these classifications remained largely neglected until the more recent time (Kaila & Vasander, 2011; Leikola, 1982; Toivonen, 2017; Vasari, 2003). Nowadays, Vainio’s thesis on plant geography is considered to have been well ahead of its time in its use of an analytical approach to explain local vegetation patterns by physicochemical soil parameters (Kaila & Vasander, 2011).

After the early papers on plant geography (Wainio, 1878a, 1878b), Vainio’s focus shifted to lichen taxonomy. In 1880 he defended his thesis “Tutkimus Cladoniain phylogenetillisestä kehityksestä” (*An investigation on the phylogenetic development of Cladonia*) which qualified him for the degree of docent (lecturer) in botany. This dissertation was likewise published in Finnish and as such, it represents the first printed discussion of phylogenetics written in Finnish. This study led Vainio to begin a world monograph of the lichen genus *Cladonia*, which was consequently published in three volumes (Wainio, 1887, 1894, 1897). In the monograph Vainio presented a natural classification of *Cladonia* based on the phylogenetic method described in his thesis (Wainio, 1880a) and further developed in the third volume of the monograph. Meanwhile, Vainio made his most important field expedition to Brazil in 1885, and on the basis of material collected during this trip and visits to herbarium in Paris published “Étude sur la classification naturelle et la morphologie des lichens du Brésil, I–II” (*A study on the natural classification and morphology of the Brazilian lichens, I–II*) (Wainio, 1890), which was written as a thesis to compete for a professorship in the Imperial Alexander University, Helsinki (now the University of Helsinki) (Ahti, 1998; Alava, 1986). The new lichen classification proposed in *Étude* was based on Schwendener’s (1868) radical idea that lichens are composite organisms composed of algae and fungi. This required a totally new classification scheme whereby Vainio classified lichens as fungi and not as an autonomous plant group, as was commonplace (Mitchell, 2015; Tibell, 1998). Furthermore, Vainio concluded that “les Lichens composent un groupe polyphylétique caracterisé par des phénomènes biologique analogues” (*the lichens form a polyphyletic group characterized by analogous biological phenomena*) (Wainio, 1890, p. XIV) – a bold statement, which took a century to become fully accepted (Tibell, 1998). These revolutionary views did not help in the competition for the professorship – quite the contrary –, and Vainio did not get the position. Official reason was his narrow scope in lichen systematics, but he was also criticized for not following his own rationale – despite claiming that lichens should be classified in the fungal system, Vainio still used the photobionts (algae) to recognize some of his taxonomic groups (Vitikainen, 1998). Personally, however, Vainio was convinced that he was not selected because of purely language-political reasons (Wainio, 1892).

After failing to get the professorship and without any university position, Vainio, who had just got married, got a position as a press censor (Linkola, 1934). This alienated him even more from the academic world and his fellow countrymen, although he maintained contacts with his international colleagues and continued his lichenological studies (Collander, 1965). Finland achieved independence and the press censorship maintained by the Russian government was lifted in 1917. Without a job Vainio got into economic difficulties, until a privately funded Finnish university was founded in Turku in 1920. Vainio not only sold his entire lichen and plant collection to the newly founded university, but at the age of 69 years also got his first academic position as a curator of the new herbarium in the University of Turku (Stenroos, 1998). Besides curating the collections he also participated in teaching and actively published numerous smaller lichenological treatments in addition to his last major study, *Lichenographica Fennica*, of which he was not able to complete all the volumes before his death in 1929.

Vainio’s lichen collection kept in the University of Turku herbarium (TUR) with thousands of type specimens has been meticulously documented (Alava, 1986, 1988, 2008) and his opinion on lichen classification has been evaluated from a modern perspective (Mitchell, 2015; Tibell, 1998). As well, his early studies on plant geography have received well-earned attention (Kaila & Vasander, 2011; Leikola, 1982; Toivonen, 2017; Vasari, 2003) and he is briefly mentioned in the accounts of the early history of Darwinism in Finland (Hällström, 1909; Leikola, 2009). But Vainio was much more Haeckelian than Darwinian and as a systematist his main theoretical interest was in the use of phylogenetic reasoning in building a natural classification. Vainio was the first phylogeneticist in Finland and he adopted an entirely phylogenetic approach to systematics already by 1880, but so far his revolutionary phylogenetic thinking has gone practically unnoticed (but see Stenroos & DePriest, 1998; Tibell, 1998). In this paper, I aim to provide an overview of his phylogenetic theory and method.

## The origins of Vainio’s phylogenetic thinking

Vainio started his studies in the university in 1870 and by 1880 had adopted an entirely phylogenetic opinion on systematics: “Descendensi-teorian kannalta ei systematiikin tehtäväksi enään tule järjestää organismia niin että ne, joilla on enimmin yhtäläisyyttä, tulevat rinnatusten, tai niin että vasta-alkavainen vähimmällä vaivalla saapi selkoa eli yleisen katsahduksen niistä; sen tehtäväksi tulee etsiä niiden geneetillistä yhteyttä, – se muuttuu, toisilla sanoilla, genealogiaksi” (*From the perspective of the theory of descent the task of systematics will no longer be to classify organisms so that the most similar ones are placed together, or so that a beginner can get an overview of the classification with the least trouble, but its task will become to search for genetic connections, – it will change, in another words, to genealogy*) (Wainio, 1880a, p. 2). The direct reference to the theory of descent lacked any citation and Vainio only cited the German translation of *The Origin of Species* as a reference to wider distributions of species relative to their varieties in his thesis. Ernst Haeckel (1834–1919) was not even mentioned. However, the practice of scientific referencing at that time differed considerably from the modern standards, for example, Charles Darwin (1809–1882) omitted most of his sources in his *Origin of Species* (de Lima Navarro & de Amorim Machado, 2020).

Vainio probably first heard about Darwin's theory from his brother-in-law and botany teacher Johan Petter Norrlin (1842–1917), possibly already in 1867. In that year, Norrlin had made an expedition to Lapland together with three other young Finnish biologists, Aukusti Juhana Mela (1846–1904, until 1876 August Johan Malmberg), Johan Axel Palmén (1845–1919) and Johan Sahlberg (1845–1920) (Lappalainen, 1956). Palmén had just obtained Darwin’s *Origin of Species* and was reading it during the expedition – and constantly talking about it with his travel companions (Lappalainen, 1959). Norrlin had previously given home-schooling to Vainio’s elder brother, often botanized with young Vainio, and later married their sister, so these two families were close (Cajander, 1942; Linkola, 1934). In 1872 Mela gave a talk “ihmisen synty” (*man’s origin*) in the Savo-Karjalainen student’s club, in which club Vainio was very active at the time as a student (Lappalainen, 1959). Mela’s talk was not influenced only by Darwin, but also by Haeckel’s *Natürliche schöpfungsgeschichte* published in 1868 (Lappalainen, 1959, p. 92). Palmén was familiar also with Haeckel’s *Generelle Morphologie der Organismen*, published in 1866, although it remains unclear when he first read Haeckel (Hällström, 1909). Later on, Palmén was in correspondence with Haeckel (Haeckel Archive, https://haeckel-briefwechsel-projekt.uni-jena.de/de) and as a professor of zoology in the University of Helsinki he belonged to the same academic circles with Vainio. Indeed, preserved letters prove that Mela, Palmén and Vainio were in contact with each other (Lappalainen, 1959, p. 203). Haeckel even visited Finland in 1897 (Olsson & Hoßfeld, 2019), but by that time Vainio had already published his phylogenetic views and there is no evidence of Vainio ever corresponding directly with Haeckel, although Vainio also travelled to Germany. Vainio's personal library kept in herbarium TUR does not include any of Haeckel’s publications, but he had a Swedish translation of *The Origin of Species* from the year 1871. Despite this, Vainio was clearly familiar with Haeckel’s writings and refers to him in his surviving lecture manuscripts.

It is clear that Vainio´s phylogenetic ideas were not inspired only by Darwin and Haeckel. He sympathised with the German school of botany and did refer to other early influential authors, most notably the Swiss-German Carl Wilhelm von Nägeli (1817–1891). Although there is again no evidence of direct communication between Vainio and Nägeli, Norrlin did meet Nägeli in Germany in 1877 (Enroth & Kukkonen, 1999, p. 443) and likely passed some of his thoughts to Vainio.

**Fig. 2:**
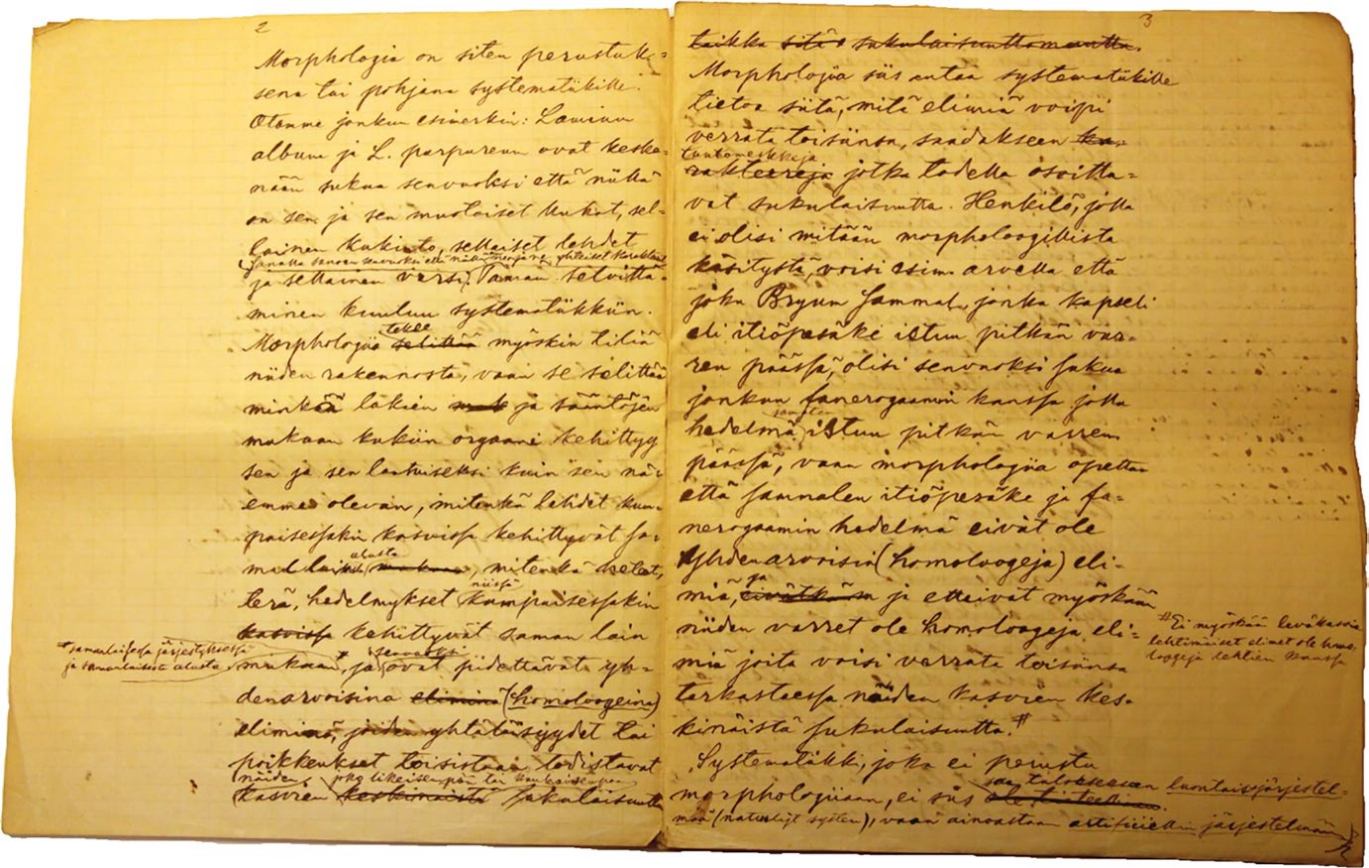
Two pages of Vainio’s handwritten and unpublished lecture “Luentoja morphologiasta”, dated in year 1882 and stored in the archives of the University of Turku. (Color figure online)

Vainio’s available letter exchange does not indicate any exchange of phylogenetic or evolutionary thoughts with his correspondents, but in addition to his published works dealing with phylogenetics (Wainio, 1880a, 1890, 1897) he has covered the topic in his lectures. One of the lectures, “kasvitieteellisestä luontais-systeemistä” (*On the natural system in botany*), which is likely the first academic lecture in botany ever given in Finnish language,^2^ is published (Wainio, 1880b). In addition to this, two of his hand written lecture manuscripts dealing with evolution and phylogenetics have partially survived in the archives of the University of Turku. These are the undated and only partially preserved lecture concerning the theory of evolution entitled as “Ch. Darwinin oppi laatujen synnystä Kasvi- ja Eläin-kunnassa” (*Ch. Darwin's theory on the origin of species in plant and animal kingdoms*) and the more phylogenetically oriented lecture “Luentoja morphologiasta” (*Lectures on morphology*) dated in year 1882 (Fig. 2). These lectures are of particular interest, because here Vainio actually refers to his sources unlike in his research texts. Apparently it was important to teach the students who the authors of original ideas were, because they were not cited in the publications. These published and unpublished works by Vainio form the basis of this review.

## The nature of evolution

Vainio’s undated lecture manuscript on Darwin’s theory, or what is left of it, deals with the history of the theory and the basic principles of evolution. It provides an overview of how natural history had evolved from purely theological explanations through Aristoteles and Linné towards evolutionary explanations. After brief descriptions of Jean-Baptiste Lamarck’s (1744–1829) work (Lamarck, 1809) and Étienne Geoffroy Saint-Hilaire (1772–1844), Vainio continues to summarise the key points of Darwin's theory. Importantly, Vainio points out that Darwin explains individual variation to arise through interaction of external (environmental) and internal factors. This matter Vainio later discussed broadly in the context of understanding the phylogenetic evolution in *Cladonia* (Wainio, 1897). Another relevant paragraph of this manuscript is the one revealing Vainio’s opinion on the purpose of science: “nykyajan tieteellisellä tutkimuksella ei ole mitään muuta tarkoitusta kuin löytää totuuden ja sitä juuri totuuden tähden” (*modern scientific research has no purpose other than to find the truth, and that only for the sake of the truth*). This is an opinion quite similar to what Matthias Jakob Schleiden (1804–1881) had presented: “Ueberall aber ist das Ideal, welches mich belebte, Wahrheit gewesen und zwar die reine und ungeschminkte Wahrheit” (*But everywhere the ideal that enlivened me was truth, and indeed the pure and unvarnished truth*) (Schleiden, 1842, p. XIV). Indeed, it seems like Vainio had built the whole lecture following Schleiden’s (1842, p. 2) instructions and Vainio did cite him elsewhere (Wainio, 1880b, p. 190). It is thus quite clear that Schleiden is one of the key figures behind Vainio’s science philosophy. Haeckel and Nägeli were likewise inspired by Schleiden and anxious to move to Jena to work with him (Farlow, 1890; Richards, 2008).

Vainio continues the lecture by describing how human welfare and the economy has improved after religious explanations were gradually replaced with a scientific world view, but concludes that improving the economy is not the highest purpose of the science; it is the search for the laws of nature that must be the ultimate goal of science. The goal of systematics is a natural classification that is consistent with the course of evolution (Wainio, 1880b). As Vainio later wrote: “Si les systêmes établis suivant des principes différents ne s’accordent pas, c’est que I’un ou I’autre ne représente pas la classification naturelle, dont il ne peut exister qu’une seule, á savoir celle qui indique l’évolution que les plantes ont réellement suivie, au cours d’un temps incalculable” (*If the systems established according to different principles do not agree, it is because one or the other does not represent the natural classification, of which there can exist only one, namely that which indicates the evolution that the plants have actually followed, over an incalculable time*) (Wainio, 1897, p. 96).

A much deeper insight into Vainio’s view on the nature of evolution can be found from his world monograph of *Cladonia* (Wainio, 1897). In the general part of the monograph there is an ample discussion of different causal agents behind different kinds of character transmutations. Vainio had previously investigated the roles of soil characteristics and climate in determining local plant communities (Wainio, 1878a) and followed Darwin (1859) in believing that environment can induce transmutations: “… les conditions exterieures dont les Cladonies subissent l´action exercent une influence importante relativement à la production des variations dans les espèces de ce genre” (… *the external conditions to which the Cladonia are subjected exert an important influence in producing variations in the species of this genus*) (Wainio, 1897, p. 134). But characters can also change by internal causes (autogenetically), and this is the case when variations cannot be attributed to the influence of the external environment – internal causes can also be ascertained through experiments (Wainio, 1897, p. 141). It is evident that the early ideas of inheritance presented by Haeckel (1868) and Nägeli (1884) strongly influenced Vainio.

According to Vainio, the environmentally induced transmutations have lower heredity than internally induced, but because internal processes always require certain external conditions and external factors provoke changes in vital internal processes (compare with Nägeli, 1884, p. 344), the external and internal drivers of evolution are always connected to a certain degree (Wainio, 1897, p. 169). Hence, environmentally caused transmutations typically occur in the population repeatedly and are ephemeral (low heredity), but the transmissibility of such characters can increase through time, i.e., they may become increasingly hereditary until their appearance is no longer dependent on external agent (Wainio, 1897, p. 164). Vainio summed up a general law: “L'évolution des variations et des espèces est déterminee par le milieu extérieur et des causes internes, or l’un ou l’autre de ces agents exerce dans certaines phases une influence prédominante, en donnant l’impulsion à la production de différents caractères” (*The evolution of variations and species is determined by the external environment and internal causes, yet one or the other of these agents exerts in certain phases a predominant influence, giving impetus to the production of different characters*) (Wainio, 1897, p. 170).

The task of systematists was to carefully study these character changes in order to find out their causing agents and this way reveal the phylogeny in a Haeckelian sense – for Haeckel, phylogeny meant the series of changes occurring in a morphological character over the evolutionary history (see Dayrat, 2003). Likewise, Vainio wrote: “lajien keskinäinen sukulaisuus ei ole toteennäytetty … siksi kuin elin elimeltä niiden phylogenetillinen kehitys on selvitetty” (*the mutual kinship of the species has not been proven … until the phylogenetic development has been clarified organ by organ*) (Wainio, 1880b, p. 24). The phylogenetic method Vainio was seeking,^3^ hence, was primarily a set of rules to determine the course of character evolution from environmentally caused characters of low heredity to internally induced characters of higher inheritance, and to avoid the pitfalls of reversals and other evolutionary processes hiding the true history. The resulting character phylogenies would then reveal the evolutionary relationships of the species, the natural classification.

## Natural classification

Vainio's published lecture on the natural classification of plants (Wainio, 1880b) dates the same year as his thesis on the phylogenetics of *Cladonia* (Wainio, 1880a). These texts are dealing with different perspectives of systematics and the thesis, actually, gives only a very brief theoretical background before entering into describing and analysing characters and their “phylogenetic development”. In the lecture, however, Vainio is discussing the nature of species and higher taxa, and describes how folk taxonomies got first replaced with idealistic classifications by authors like Carl von Linné (1707–1778) and Elias Fries (1794–1878), until a new “inductive” approach to plant morphology and botany took over by authors like Schleiden, Nägeli, and Wilhelm Hofmeister (1824–1877).

The lecture ends with criticism of his contemporary systematics. Despite accepting the theory of evolution, systematists still continued the old descriptive tradition and tended to avoid the more intellectually demanding study of organizing the empirical observations into a natural system: “… systematiikissa niin sitkeästi säilyvä inkonsekventti deskriptiivillinen suunta, joka ei ota vaivakseen järjestää induktiivillä tiellä tekemiä havainnoitaan tieteelliseen kokonaisuuteen, systeemiin, vaan hyväksyen uudemman luonnontutkinnon, lajien muuttuvaisuuteen perustuvia päätelmiä, inkonsekventisti kuitenkin on sen metodia noudattamatta deskriptivillisessä laji-tutkimuksissaan ja tyytyy paljaiden faktain muistiinpanoon sellaisina kuin ne ilman tarkempaa ajattelemista esiytyvät heille” (… *the inconsistent descriptive school that is so persistent in systematics, does not bother to organize its observations in an inductive way into a scientific whole, a system, but despite accepting the newer natural science's conclusions based on transmutation of species, still inconsistently does not follow its method in descriptive species-investigations, but settle for recording bare facts as they appear without further thought*) (Wainio, 1880b, p. 191). In contrast to the old school, Vainio follows Nägeli and the German inductive school of botany and, once again, the text is echoing Schleiden (1842, p. 9). Given the references to Schleiden, Nägeli, and Hofmeister in this lecture, and direct citation of Julius Sachs (1832–1897; Sachs, 1874) in his morphology lecture, we can affirm that Vainio’s ideas were strongly rooted in the German tradition of botany (see Cittadino, 2002). All these German authors were also proponents of Darwin's theory (Glick, 1988).

This lecture reveals many of the sources behind Vainio’s thinking. It is noteworthy that Haeckel is mentioned only in passing. Vainio refers to Hofmeister’s (1851) embryological studies in higher cryptogams and conifers and explains how the method Hofmeister used “… on konsekventti descendenssiteoriasta ja jota Haeckel sittemmin nimitti phylogenetilliseksi metodiksi” (… *is consistent with the theory of descendance and was later called the phylogenetic method by Haeckel*) (Wainio, 1880b, p. 190). Vainio did not consider Haeckel as the primary author in the field of phylogenetics.

One important aspect of the lecture on natural classification is the discussion on the nature of species and higher taxa. Vainio did not dwell too deep into the nature of species in the lecture (but did so later, see below), but simply stated that those individuals which are connected to each other by intermediates form a species. This is the same phenotypic definition of species given by Darwin (1859, p. 485). Because individuals of the same species share so many characters and differ only so little, even amateurs can easily recognise species. Higher taxa are not so easily distinguished, because more of their commonly inherited characters have been further modified. This is only because more time has passed since the common ancestor: “Kun kauan aikaa on kulunut niiden erkanemisesta yhteisistä vanhemmistaan, ovat niiden yhteiset perinnölliset omaisuudet niin ehtineet kadota myöhemmin tulleiden erillaisuuksien sekaan, että ne monasti enään verrattain vähemmin pistävät silmään” (*After long time has passed since their separation from their common parents, their common inherited characters have disappeared among the differences that came later, so that they are often relatively less conspicuous*).

Therefore, higher taxa are just as natural as species:”Selvää on, että luonnossa joko löytyy paljaastaan indiviidejä, jolloin niinhyvin lajit kuin suvut, heimot, parvet ovat mielivaltaisia käsitteitä, joiden alkuperäisellä yhdenmukaisuudella ei ole mitään luonnollista selitystä, taikka on sekä lajien että parvien yhtäläisyys selitettävä niiden yhteisen syntyperän kautta. Tosin samaan lajiin kuuluvain indiviidien nykyinen yhtäläisyys on selitettävä niiden veriheimolaisuuden kautta, kun ne ovat syntyneet keskinäisessä pariutumisessa, vaan myöskin alkuansakinhan niiden on täytynyt olla yhtäläisiä, sillä muutoinhan ne eivät olisi voineet hedelmöittää toisiaan. Siten myöskin samaan lajiin kuuluvain indiviidien alkuperäinen yhtäläisyys on saman probleemin alainen kuin sukujen, heimojen ja luokkain yhdenmukaisuus” (*It is clear that there are either bare individuals in nature, in which case species as well as genera, families and categories*^4^* are arbitrary concepts whose original uniformity has no natural explanation, or the similarities of both species and categories must be explained through their common origin. Admittedly, the present similarity of individuals belonging to the same species must be explained by their blood-kinship, as they mate with each other to give birth, but even so, in the beginning they also must have been similar, for otherwise they could not have fertilized each other. Thus, the original uniformity of individuals belonging to the same species is subject to the same problem as the uniformity of genera, families, and classes*) (Wainio, 1880b, pp. 185–186). Here, Vainio follows again the view of Nägeli, who argued that species as well as higher taxa are not abstractions of human mind but real entities due to their common origin: “Der Schwerpunkt der naturgeschichtlichen Betrachtung liegt nicht mehr in der Species, sondern darin, dass jede systematische Kategorie als eine natürliche Einheit gefasst wird, welche den Durchgangspunkt einer grossen entwickelungsgeschichtlichen Bewegung darstellt. Die Gattung und die höhern Begriffe sind keine Abstractionen sondern concrete Dinge” (*The focus of the natural-historical consideration is no longer in the species, but in the fact that each systematic category is conceived as a natural unit, which represents the point of passage of a large evolutionary movement. The genus and the higher concepts are not abstractions but concrete things, complexes of related forms that have a common origin*) (Nägeli, 1865, p. 32). Haeckel (1868, p. 39) supported similar view “Weiterhin müssten dann aber, der Abstammungs-Lehre entsprechend, auch alle verschiedenen Gattungen einer und derselben Ordnung von einer einzigen gemeinschaftlichen Urform abstammen, und ebenso endlich alle Ordnungen einer Classe von einer einzigen Stammform” (*all the different genera of one and the same order ought also to be descended from one common primary ancestor, and so, in like manner, all orders of a class from a single primary form*). Haeckel was quite specific, however, that ranking of species as well as higher taxa is by necessity arbitrary (Haeckel, 1866, p. 378; see also Rieppel, 2011a), but Vainio does not seem to follow Haeckel in this respect. Darwin (1859, p. 422) held the view that ranking must express the degrees of modification that has taken place during the course of evolution and Vainio seemingly had similar opinion; higher taxa share fewer commonly inherited characters. These are characters that persist over longer evolutionary times than others, and are therefore useful to recognise taxa at different ranks: “Tärkeitä poikkeuksia ovat varsinkin sellaiset, jotka koskevat heimo-, parvi-, suku-, ryhmä- ja laji-karakteereja” (*Important exceptions are especially those concerning the family, category, genus, group and species characters*) (Wainio, 1880a, p. 39). Vainio’s opinion that taxa can be objectively ranked after appropriate character analysis seems to be much closer to Nägeli than Haeckel (see Rieppel, 2011a).

In fact, the *Cladonia* monograph (Wainio, 1897) can even be seen as an answer to Nägeli’s wish: “Der künftigen Systematik wird er [Begriff der Species] eine wissenschaftliche Kategorie sein, für die es bestimmte in der Natur zu beobachtende, durch das Experiment zu prüfende Merkmale giebt” (*In future systematics it* [the concept of species] *will be a scientific category for which there are certain characteristics that can be observed in nature and tested by experiment*) (Nägeli, 1865, p. 33). For Vainio, species was not an abstraction, but rather a concept defined in a way that sounds peculiarly similar to some modern phylogenetic species concepts, as we shall see.

## Vainio’s method

The first step towards a natural classification was to determine if characters under study were homologous or analogous, i.e., if the characters were related through blood kinship, or if they were similar despite independent origin. Analogous characters were too uncertain to be used in reconstructing natural classifications (Wainio, 1897, p. 89). In his morphology lecture, Vainio stated that homologs can be detected through studies of either ontogeny or comparative morphology. Citing Haeckel, Vainio considered ontogeny more important, because “kuta nuoremmassa kehitystilassa sitävastoin elimet ovat, sitä tarkemmin ne todennäköisesti kuvastavat elimien phylogenetillista kehitystä, alkuperäistä tilaa” (*the younger the organs are in their developmental stage, the more accurately they probably reflect the phylogenetic development of the organs, the original state*). When a character differed between the adults of different species, ontogeny revealed the evolutionary series of character state transformations – it recapitulated the character phylogeny. Hence, ontogeny revealed the polarity of character state transformations, or metamorphoses^5^ in Vainio’s vocabulary. For example, on the basis of ontogeny Vainio concluded that horizontal primary thallus is a primitive character in *Cladonia*, from which podetia grow as secondary thallus formation (Wainio, 1880a, p. 7). Hence, the closest relatives of *Cladonia* should be searched among those lichens sharing the character state primitive in *Cladonia* – horizontal thallus (Wainio, 1880a, p. 7). Later, perhaps due to criticism of the recapitulation theory, Vainio downplayed the role of ontogeny by noting that “En effet, ce n’est pas une loi sans exception que le développement ontogénétique répète l’évolution phylogénétique” (*It is not a law without exception that ontogenetic development repeats phylogenetic evolution*) (Wainio, 1897, p. 91).

Thus, ontogeny revealed the character phylogeny, and the main interest was to detect the primitive character states, because by combining these “propriétés inférieures” (*lower properties*) it was possible to reconstruct ancestral types (“prototype collectif”). Vainio listed numerous characters presenting lower and higher degrees of evolution in *Cladonia*, but noted that the lower states of different organs do not necessarily occur together in the same species; “Cependant il faut remarquer que le type collectif des aïeux des Cladonies ainsi reconstruit est fictif à un certain degré, selon toute vraisemblance. Il n’est point nécessaire que toutes ces propriétés se soient jamais trouvées unies dans la même plante” (*However, it should be noted that the collective type of the ancestors of the Cladonias thus reconstructed is fictitious to a certain degree, in all likelihood. It is not necessary that all these properties have ever been found united in the same plant*) (Wainio, 1897, p. 84). Species, therefore, were a mosaic of primitive and derived characters (Wainio, 1897, p. 87). From this observation arose a key idea, the full value of which was not understood until much later.

This idea was rather clearly presented already in Vainio’s earlier thesis: “Toisintojen, alilajien ja muiden nuorempain lajien suhteen on descendensi-teoriaan perustavalla systematiikilla tarkoitusperänä osoittaa, josko ne ovat toisistaan kehittyneet ja missä järjestyksessä se on tapahtunut. On senvuoksi tarkastaminen, josko parven eli lajin j.n.e. muodoista joku on pidettävä päämuotona, josta toiset eli joku toinen toisinto (laji) on kehittynyt, tai josko ne ovat ainoastaan rinnakkaistoisintoja (lajeja), jotka johtuvat jo kuolleista päämuodoista.” (*Regarding variations, subspecies and other younger species, the purpose of the systematics relying on the theory of descent is to show whether they have developed from each other and in what order it has happened. It is therefore necessary to find out whether one of the forms of the category, i.e. species *etc*., must be considered the main form from which the others have developed, or are they only parallel variations (species) descending from already extinct main forms*) (Wainio, 1880a, pp. 36–37). The same principle applies to taxa at any rank, and the question is whether a living taxon is ancestral to another, or if parallel taxa have originated from extinct forms. It remains arguably somewhat unclear if Vainio´s “parallel variations” can be taken as sister groups, but the *Cladonia* monograph later clarifies that he indeed meant sister groups in a truly Hennigian sense. In the monograph Vainio first explains that “Beaucoup d’espèces voisines paraissent, à l’instar des espèces plus éloignées, émaner d’ancètres communs appartenant à une espèce disparue. Or certaines espèces revètant un caractère qui représente une différenciation supérieure à celle d’une autre espèce voisine sont peut-ètre développées de cette espèce inférieure. Puisqu'il manque des transitions directes entre elles, il parait cependant impossible de le prouver d’une facon certaine, car il se peut aussi que ces espèces voisines soient développées d’une manière analogue d’une espèce disparue.” (*Many neighbouring species seem, like more distant species, to emanate from common ancestors belonging to an extinct species. But some species with a character which represents a superior differentiation from that of another neighbouring species are perhaps developed from this inferior species. Since there are no direct transitions between them, however, it seems impossible to prove it with certainty, because it is also possible that these neighbouring species were developed from an extinct species in analogous way*) (Wainio, 1897, p. 161). However, a crossing of specializations (see Rieppel, 2020) confirms a true sister relationship: “Lorsqu'une espèce diffère d’une espèce voisine par des caractères combinés dont I’un représente un état ancestral de ces espèces voisines, tandis qu’un autre caractère indique une évolution autonome que I’autre espèce n’a pas partagée, cette circonstance permet de conclure qu’elles ne sont pas développées I’une de I’autre, mais d’ancètres communs.” (*When a species differs from a neighbouring species by combined characters one of which represents an ancestral state of these neighbouring species, while another character indicates an autonomous evolution which the other species did not share, this circumstance leads to the conclusion that they are not developed from each other, but from common ancestors*) (Wainio, 1897, p. 161).

Much later, Willi Hennig (1913–1976) re-focused phylogenetics to specifically search for sister relationships instead of ancestors. The paradigm change began with the notion of sister relationships as revealed by crossing of specializations (Hennig, 1966, p. 194). Rieppel (2020) has investigated the history of the idea and found that it has been variedly attributed to Dollo (1895), Gegenbaur (1898), or Abel (e.g. 1913). Hennig cited Abel (1912), but since Vainio seems to have been aware of the basic idea already in 1880, it most likely has an earlier origin.

## Species and infraspecific taxa

Vainio has become infamous for his application of numerous infraspecific ranks in *Cladonia* and the use of various terms and symbols to refer to these ranks (Alava, 1988). How the different symbols should be interpreted, and indeed, what was the real concept of his infraspecific taxa, have remained a source of confusion, but are central issues in understanding Vainio's phylogenetic method. The different infraspecific ranks reflected the various stages of phylogenetic development from environmentally induced morphological variations to increasing hereditary and ultimately fixed character sets of true species. Hence, the infraspecific classification of *Cladonia* was the manifestation of the ancestor–descendant relationships at the most intimate level, the true genealogy inferred from the character metamorphoses.

**Fig. 3:**
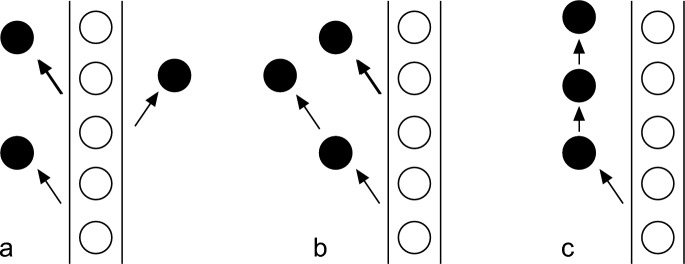
Schematic interpretation of Vainio’s terminology. A black form is produced from the white main form: **a** black form originates repeatedly from the white form, but not from any other form, it is therefore *polygenic* but *monophyletic*; **b** black form is formed repeatedly from the white form and is therefore *polygenic*, but it also reproduces itself and therefore originates from two different forms and is *polyphyletic*; **c** black form is fully constant and after its origination arises only from itself, it is therefore *monogenic*

The descriptive part of Vainio’s *Cladonia* monograph was “basée principalement sur l’affinité des espèces” (*based mainly on the affinity of species*), whereas the general part presented a phylogenetic classification, which, according to Vainio, was fully compatible with the affinity-based system (Wainio, 1897, p. 96). These two systems used different symbols for infraspecific ranks, but the “Schema” at the end of the general part linked these systems (Wainio, 1897, p. 222). Here, the lowest rank was named as “modificatio inconstans statione product” (*inconstant modification produced by the station*). These were locally and repeatedly occurring modifications of the main form, caused mainly by the unusual moisture or solar radiation of the local growing site, the station. These character changes had low heredity, although higher than irregular anomalies, and were of polyphyletic and polygenic origin (Wainio, 1897, p. 157). Vainio gave a definition for his terminology: “Une espèce ou variation qui émane de plusieurs espèces ou variations différentes est polyphylétique; si elle se produit plusieurs fois d’une autre espèce ou variation elle est polygène. Une variation est donc monogène si elle ne s’engendre plus d'une autre variation, mais se produit seulement, par hérédité, de la même variation” (*A species or variation that arises from several different species or variations is polyphyletic; if it is produced several times from another species or variation it is polygenic. A variation is therefore monogenic if it is no longer generated from another variation, but is produced only, by heredity, from the same variation*) (Wainio, 1897, p. 117). In contrast, characters (or taxa; Vainio applied these terms to both taxa and characters) were monophyletic if they originated from a single taxon^6^ (Fig. 3). Haeckel coined the terms monophyletic and polyphyletic but left them somewhat ambiguous (Rieppel, 2011b; Vanderlaan et al., 2013). Vainio, being a meticulous worker, provided his definitions that may be of interest in understanding how the phylogenetic classifications were seen in the late nineteenth century.

Vainio considered it easy to determine the degree of heredity of different characters in *Cladonia*, because they form tufts in which different forms often co-occurred, revealing their repeated origination from the ancestral type (Wainio, 1897, p. 155). In many other taxa the heredity was more complicated to reveal, but could still be done by observing the variability under different external conditions. The approach Vainio outlined is remarkably similar with the one described by Nägeli (1872), who had investigated similar problems in the Alps with a focus in *Hieracium*. It should be pointed out that Norrlin, the likely middleman between Nägeli and Vainio, was likewise a *Hieracium* specialist, and that Vainio himself visited Switzerland in 1882 and 1884 and collected, among other samples, a number of *Hieracium* specimens from the Alps.

The next rank above the modification was “forma autogenetica inconstans” (*inconstant autogenetic form*). These forms had become hereditary and were able to autogenetically maintain themselves, yet remained inconstant and repeatedly re-occurred from the ancestral main form, or often reverted back to it (Wainio, 1897). Inconstant forms often represented atavism (reappearance of the primitive character state), and therefore were important in detecting the order of character state changes. Other forms of evolutionary change included anomalies, hybrids, and normal autogenesis (either progressive or regressive) characterized by properties that constitute a differentiation from the normal state (Wainio, 1880a, 1897).

Over time, due to developing heredity, the constancy of characters may increase, until they appear regardless the environmental conditions and no longer occur spontaneously. Such characters are typically adaptive (beneficial to the plant), and taxa possessing such constant characters merit to be ranked as “varietas constantior” (*constant variety*) (Wainio, 1897, p. 160). Varieties were mostly monogenic, yet polygenic to a greater degree than the subspecies.

**Fig. 4:**
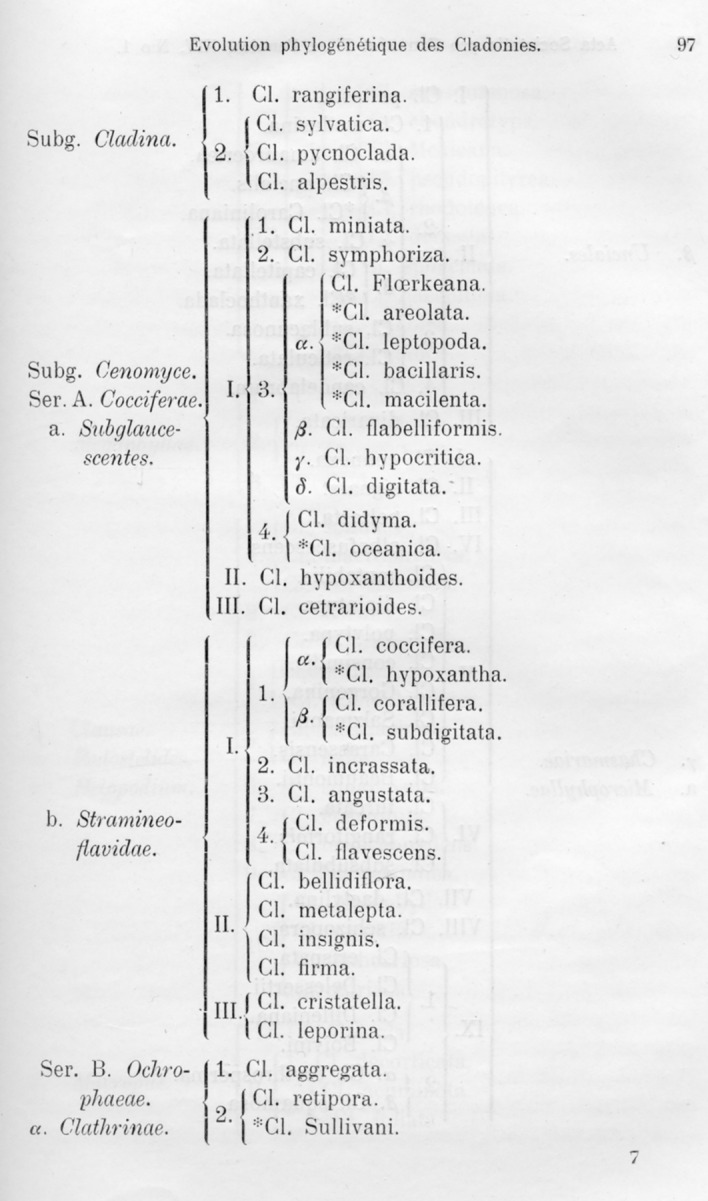
Part of the “table” summarizing Vainio’s idea of the phylogenetic evolution of *Cladonia*. This is the only tree-like depiction Vainio ever published. Subspecies are marked with an asterisk (*) and they are placed under their main species. The ranks below subspecies are ignored in this summary, but are listed in the systematic “Schema” (from Vainio, 1897: 97)

The subspecies (denoted by * in the monograph, just like the other sub-ranks, e.g. subforma) Vainio defined as mainly monogenic with a highly developed heredity, but in exceptional cases they could still repeatedly emerge from their primary species (Wainio, 1897, p. 163). Subspecies typically had a combination of characters of different age; some of which had reached hereditary and become constant, while more recent characters still varied and were polygenic. These variable characters linked the subspecies with their primary species through transitional intermediates that could occur in divergent environments (Wainio, 1897, p. 160). The lack of intermediates, or in the other words, the fixation of the polymorphic characters in such a way that offspring always show characters similar to their parents, defined the species rank (Wainio, 1897, p. 163). Hence, species were strictly monogenic and their characteristics were constant and induced by internal processes in contrast to infrageneric taxa, which were repeatedly produced by externally induced character transformations of low heredity and a tendency to revert back to ancestral state. Basically, Vainio was talking about the fixation of apomorphy through extinction of plesiomorphy in a polymorphic character. Vainio pointed out that in the case of closely related taxa, it is difficult to know if they are true species (no intermediates bridging them) or subspecies, as it may be that intermediates occur in some yet unstudied location (Wainio, 1897, p. 161). The peculiar theory of inheritance apart, Vainio’s conception of species is remarkable similar to the character-based phylogenetic species concepts that emerged hundred years later (see Nixon & Wheeler, 1990). Indeed, Nixon and Wheeler (1990) depicted species as the line separating tokogenic patterns of variable traits and population biology from phylogenetic patterns and constant characters, and Vainio similarly excluded the ranks below subspecies from his “phylogenetic table” (Fig. 4), the only tree-like representation of phylogeny he ever published. Only after he had summarized the phylogenetic evolution of *Cladonia* with the phylogenetic table, he continued with the “Variabilité des espèces”. The origins of (sub) species and higher taxa were subject to the same problem and key to the genealogy, whereas study of the lower taxa represented a character analysis with an aim to reveal the primitive and derived character states of the species, and the origin of species through the fixation of polymorphism.

## Discussion

Vainio resembled Haeckel in that he was also politically active, generally unwilling to compromise, and acquired many opponents (Willmann, 2003). For Vainio this meant exclusion from an academic career, but he still managed to become a leading lichenologist of his time, and introduced phylogenetic thinking to Finland. He was contemporary with the early development of phylogenetics in Germany and deeply interested in it, yet, he apparently never directly communicated with the German phylogeneticists. Despite being an outsider, he knew the German botanical school inside out and was well versed in the latest phylogenetic literature. Because of this view from outside, Vainio’s discussions on the phylogenetics can be considered as an excellent historical source for understanding the development of early phylogenetic thinking at the end of the nineteenth century.

Unlike several other early phylogeneticists, Vainio did not study fossils but living species. Although most of his studies represent descriptive taxonomy, he had a clear desire to develop a formal method for constructing a phylogenetic lichen classification. In this respect, Vainio was a true pioneer, because Haeckel, for example, never presented any kind of phylogenetic method (Willmann, 2003). The clear effort to create an exact method was not at all exceptional for Vainio, as he had already earlier applied a mathematical approach to community ecological studies of plants, being the first in Finland also in this respect. The desire for exact method was probably natural for Vainio, but Nägeli’s strongly mathematically oriented approach to biology (see Dröscher, 2015) certainly encouraged him as well. All in all, it seems that Nägeli may have been even more important to Vainio’s phylogenetic thinking than Haeckel, but in any case it is clear that Vainio did not see Haeckel as the only, or necessarily even the most important developer of phylogenetics. Historians of science may have overemphasized Haeckel's role in the early development of phylogenetics, as he published numerous books on the topic and furiously pursued phylogenetic thinking, which was nevertheless simultaneously developed also by other, less vocal authors.

Vainio’s ideas were clearly derived from Darwin, Nägeli and Haeckel, but nevertheless he differed from all of them and developed an unique approach to phylogenetic systematics. Already in his early thesis he disputed some of Darwin’s views (Wainio, 1880a, p. 37). Unlike Haeckel, he did not consider species to be arbitrary, but was of the opinion that phylogenetic research could objectively define species, and unlike Nägeli (1884), he believed that all higher taxa (or plants, at least) form a monophyletic group.^7^ For Vainio, species evolved from the lower forms by fixing of characters in the course of phylogenetic development through increasing character inheritance and extinction of plesiomorphies. Constant characters and monogenic origination strictly from the members of the same form only defined the species category, that was equally natural as all the higher taxa. Lower ranks were inherently different, yet relevant for understanding the order of character state changes and the fixation of polymorphism during the speciation process. Vainio did distinguish analogues from homologues and derived character states from the primitive, and he was fully aware of character conflict. He was even familiar with the concept of sister groups and how the crossing of specializations revealed them, but of course, without understanding the true value of sister relationships. Instead, they were rather a source of error when searching for direct ancestor–descendant relationships, which was the real goal of the phylogenetic systematics. In addition to the phylogenetic method itself, Vainio amply discussed phylogenetic biogeography (Wainio, 1897), but this topic is beyond the scope of the present paper.

Since Vainio worked mainly outside the academia he had no students. An exception was Veli Räsänen (1888–1953), who specialized on lichens as a student in the University of Turku under Vainio’s guidance. Räsänen continued the legacy of Finnish lichenology after Vainio, but never discussed phylogenetics and did his research outside the academia. His contributions towards a “natural” lichen classification were ignored (Mitchell, 2015). Although Vainio was a highly regarded lichenologist and his species-level taxonomy is considered outstanding (Ahti, 1998), his phylogenetic views apparently did not spread anywhere (but note the commentary by Choisy, 1927).

Nevertheless, some other Finnish systematists adopted a phylogenetic approach early on. These included Enzio Reuter (1867–1951), who defended his dissertation on the phylogenetic systematics of butterflies in 1896 (Reuter, 1896), and his elder brother Odo Morannel Reuter (1850–1913), who attempted to study the phylogenetics of hemiptera in his old age (Leikola, 2009). A mycologist Johan Ivar Liro (1872–1943, until 1906 Lindroth) presented a phylogenetic diagram of rust fungi relationships in 1902 (Lindroth, 1902) and a zoologist, later a researcher in the history of biology, Erik Nordenskiöld (1872–1933) published a phylogenetic tree of mites in 1898 (Nordenskiöld, 1898). However, none of these authors discussed the theory of phylogenetics as deeply as Vainio, nor presented a method as the basis of their phylogenetic considerations. It remains unclear to what extent Vainio may have inspired them, or whether they adopted their phylogenetic ideas directly from Germany, to which Finnish researchers were very close at the time. None of them cited Vainio or seem to have followed his method, but Vainio was already an academic outcast and persona non grata within the Finnish academia, and probably would have been excluded from the references even if he had had some influence. The early Finnish authors were keen to adopt the latest phylogenetic ideas, but there is no evidence about their contribution to the development of the field. A few decades later a wave of new Hennigian phylogenetics spread to Scandinavia, and also to Finland (Seberg et al., 2016).

## Footnotes

1 Vainio changed his original Swedish surname Lang to Finnish Wainio in 1876 and later adopted modern Finnish writing style Vainio. The fennicisation of foreign names began in the Finnish nationalist student circles. Vainio had been an active advocate of the fennicisation, perhaps even the initiator, but due to illness he could not change his own name among the very first name changers (Linkola, K. unpublished statement to the Faculty of Natural Sciences, University of Turku, 22.2.1923).

2 The first academic botany lecture in Finnish language was given by Vainio in the fall of 1880, but the topic of that lecture has not been reported (Collander, 1965, p. 90). It is quite possible that the published lecture is the one.

3 "… olemme ainoastaan tahtoneet elin elimeltä, tunnusmerkistä tunnusmerkkiin, seurata Cladoniain alkumuodon kehitystä nykyisiin uusimpiin muotoihinsa, samalla tutustuaksemme siihen metodiin, johon kasvi-genealogillinen tutkimus on perustettava, ja, missä metodi on ollut tuntematon, sitä etsiäksemme" (… *we have only wished to trace, organ by organ, from character to character, the development of the ancestral form of the Cladonias to its present latest forms, to become at the same time acquainted with the method on which plant-genealogical research must be founded, and, where the method has been unknown, to seek it*) (Wainio, 1880a, p. 2).

4 Here I translate the Finnish word "parvi" (a word that means a cluster, or flock, school, pack, or other grouping of usually animals) as "category" following Nägeli's (1865) "Kategorie", a probable source of Vainio. Vainio gave an indirect definition for his "parvi": "… sukujen, heimojen ja muiden ylhäisempien parvien…" (… *genera, families and other higher categories…*) (Wainio, 1880b, p. 185) and later on: "luonnossa oli olemassa myöskin muita parvia kuin paljastaan lajeja" (*there were in the nature categories other than just species*) (Wainio, 1880b, p. 186). Vainio used the word "parvi" also for unranked categories; "… erythrocarpit Cladoniat ovat … epäilemättömästi luonnollinen parvi, joka eroaa muista Cladonioista…" (… *erythrocarpean Cladonias are … without doubt a natural category that differs from other Cladonias*…) (Wainio, 1880a, p. 25). In modern times, we would simply use the word "clade" or perhaps "taxon". It is noteworthy that there is still no Finnish word for "clade" unless we adopt Vainio's "parvi" in this meaning (unlike "parvi", the sometimes used word "kladi" can hardly be considered proper Finnish. "Taxon", on the other hand, smoothly and consistently translates as "taksoni" in modern Finnish).

5 Vainio defined metamorphosis in his morphology lecture: "metamorphoosi on homoloogien eli samansukuisten elinten erilaatuinen kehitys eri tarkoituksiin" (*metamorphosis is the different development of homologous, i.e. kinship, organs for different purposes*). The concept of metamorphosis originated with Goethe (1790).

6 "Si l'on admet une origine monophylétique aux podétions, c'est-à-dire que les podétions se soient formés primitivement dans une seule espèce, qui ensuite a engendré les autres espèces pourvues de podétions…" (*If we admit a monophyletic origin of the podetions, that is to say that the podetions were formed primitively in a single species, which then engendered the other species with podetions…*) and "Si, au contraire, on suppose une naissance polyphylétique aux podétions, c'est-à-dire qu'ils aient apparu séparément dans plusieurs espèces voisines…" (*If, on the contrary, we suppose a polyphyletic origin of the podetions, that is to say that they appeared separately in several neighbouring species…*) (Wainio, 1897, p. 86). Characters that were polyphyletic in general could still be monophyletic for smaller groups: "Cela n'empêche pas que les caractères en question soient monophylétiques dans les groupes où ils sont constants" (*This does not prevent the characters in question from being monophyletic in the groups where they are constant*) (Wainio, 1897, p. 90). Vainio applied the terms polyphyletic, monophyletic, polygenic and monogenic to both characters and taxa. It seems that Vainio considered paraphyly as monophyly.

7 In a foonote of his morphology lecture Vainio clearly expressed his opinion, that even if polyphyletic origin of simple life forms cannot be ruled out, the higher organisms must share a common ancestry with the highest probability. It remains unclear if he still accepted the possibility for independent origins of monophyletic plants and animals, for example, in the same way as Haeckel (Rieppel, 2011b).
